# High-resolution Imaging of pH in Alkaline Sediments and Water Based on a New Rapid Response Fluorescent Planar Optode

**DOI:** 10.1038/srep26417

**Published:** 2016-05-20

**Authors:** Chao Han, Lei Yao, Di Xu, Xianchuan Xie, Chaosheng Zhang

**Affiliations:** 1 State Key Laboratory of Lake Science and Environment, Nanjing Institute of Geography and Limnology, Chinese Academy of Sciences, Nanjing 210008, China; 2 State Key Laboratory of Pollution Control and Resource Reuse, Center for Hydroscience Research, School of the Environment, Nanjing University, Nanjing 210093, China; 3 GIS Centre, Ryan Institute and School of Geography and Archaeology, National University of Ireland, Galway, Ireland

## Abstract

A new dual-lumophore optical sensor combined with a robust RGB referencing method was developed for two-dimensional (2D) pH imaging in alkaline sediments and water. The pH sensor film consisted of a proton-permeable polymer (PVC) in which two dyes with different pH sensitivities and emission colors: (1) chloro phenyl imino propenyl aniline (CPIPA) and (2) the coumarin dye Macrolex^®^ fluorescence yellow 10 GN (MFY-10 GN) were entrapped. Calibration experiments revealed the typical sigmoid function and temperature dependencies. This sensor featured high sensitivity and fast response over the alkaline working ranges from pH 7.5 to pH 10.5. Cross-sensitivity towards ionic strength (IS) was found to be negligible for freshwater when IS <0.1 M. The sensor had a spatial resolution of approximately 22 μm and aresponse time of <120 s when going from pH 7.0 to 9.0. The feasibility of the sensor was demonstrated using the pH microelectrode. An example of pH image obtained in the natrual freshwater sediment and water associated with the photosynthesis of *Vallisneria spiral* species was also presented, suggesting that the sensor held great promise for the field applications.

pH is one of the key biogeochemical parameters reflecting the thermodynamic state of acid-based processes and overall balances between multiple reaction and transport processes within natural environments^1^,^2^,^3^. Various biogeochemical processes in the sediment including heterotrophic respiration, photosynthesis, metabolism, sedimentation and dissolution, etc., which are all involved in the protonic reactions, can cause a steep pH gradient in the vicinity of the sediment-water interface (SWI) on a submillimeter scale^4^,^5^. Accordingly, an accurate detection of the distribution and variation of pH is important for a better understanding of the biological, chemical, and physical processes in the sediments. Furthermore, such detection can reveal more relevant information about these progresses.

Owing to the importance of pH measurement, a series of analytical methods have been developed in different matrices. Most of the well-established tools for pH measurement at a high spatial resolution are either pH micro-electrodes or fiber optical sensors, which have been widely used with great success under lab or field conditions^6^,^7^. However, obvious drawbacks of micro-electrodes include fragility, high cost, and time-consuming data collection with tedious operations. Optical fiber sensors have served as a proven alternative to micro-electrode-based methods and have overcome many of these problems. Similar to electrodes, optical fiber sensors are limited to single-point measurements, which can only provide a simple vertical pH profile. Neither of them are satisfactory for synoptically resolving complex compositional patterns in spatially heterogeneous sediments^2^,^8^.

Recently, there is an increasing interest in the planar optode technique. This technique enables a non-invasive quantitative mapping of 2D analyte distribution at various heterogeneous matrices with unique advantages over other methods, including excellent spatial resolution, high selectivity and sensitivity, low cost, simple preparation and measurement procedures, and relatively fast response, which will consequently contribute to the successful elucidation of biogeochemical processes^9^,^10^. A planar optode setup consists of a sensing layer containing an analyte-sensitive dye immobilized in an analyte-permeable polymer or sol-gel matrices coated onto a support foil (e.g., PET, Mylar) and a camera-based imaging system (see Supplementary information, Fig. S1). Upon excitation (commonly by LED or UV/Xenon light), luminescence intensity or lifetime emitted from the sensing layer is recorded by the imaging system. Over the last decade, numerous studies have used the planar optodes for the measurements of various biogeochemical parameters such as O_2_^11^, CO_2_^12^, pH^13^, etc. Hulth^1^ reported the first optical pH planar sensor, using the indicator, 8-hydroxy−1,3,6-pyrenetrisulfonic acid trisodium salt (HPTS), for gaining insights into the dynamics and distribution of pH at the SWI in a marine environment^1^. However, sensor drawbacks such as relatively soft, fragile, and low pH dynamic range (pH 5.4 ~ 7.4) limit its applications^14^.Thereafter, Zhu *et al*.^2^ modified the immobilization of HTPS by covalent bonding of the fluorophore to the transparent poly(vinyl alcohol) membrane, which was suitable for various matrices with a dynamic range from pH 6.0 to 8.0^2^. By synthesizing a new pH indicator or modifying the immobilization, a large number of pH planar optodes have been developed to visualize pH dynamics of the rhizosphere^15^, living cells^16^, marine sedimentdiagenesis^2^,^5^, medical diagnoses^17^ and microbial mats^18^.

There are many alkalineaquatic ecosystems in nature, such as the thousands of lakes in Hulun Lakes, Tibetan Plateau, China and Nebraska Sand Hills, USA with pH values ranging from 8.0 to 10.0^19^,^20^. Past and current researches confirm that the outbreak of cyanobacterial blooms causes an elevation in water pH between 8.0 and 9.5 in some lakes which can last for several weeks^21^. The hypereutrophic systems with high primary productivity also experience a remarkable pH elevation as high as 10.0 due to the disequilibrium of pH-buffering system induced by strong photosynthesis^22^. Additionally, the photosynthesis of submerged plants such as *Chara vulgaris Linn* and *Vallisneria spiralsv*can strongly increase the pH toa high alkaline level (pH 9.0 ~ 10.0)^23^. Whereas a number of pH optodes were described, and most of those reported pH ranges are acid or near neutral, only a few pH sensors have currently been set up to image the pH in inalkaline sediments. For example, some researchers reported pH optodes for marine monitoring empolying lipophilic carboxyfluorescein derivatives, i.e., 2′,7′-dihexyl–5(6)-N-octadecyl-carboxamidofluorescein (DHFA) and 2′,7′-dihexyl–5(6)-Noctadecyl-carboxamidofluorescein ethyl ester (DHFAE) as the indictor and lifetime imaging approaches, which showed better sensitivity and accuracy in a broad range between pH 7.0 and 9.3^14^,^24^. Aigner *et al*.^25^ described a series pH optodes by selecting three 1,4-diketopyrrolo-[3,4-c]pyrrole derivatives (DPPs) as fluorescent indicators and different polymer hydrogels as the matrix^25^. The operational pH can be tuned over a wide range between pH 5.0 and 12.0 by combining those independent DPPs-based sensors. Those sensors generally require sophisiticated, expensive analytical devices or relatively time-consuming synthesis and tedious purifying procedures for reagents and thus, are not available in many analytical laboratories. As a consequence, design and development of more accessible fluorescent pH sensors for alkaline aquatic ecosystems are still ongoing.

In this study, we propose a new sensor film with sensitive fluorescence response to pH variation from pH 7.5 to pH 10.5 A series of validation experiments were conducted to evaluate the performance of the pH sensor under the laboratory conditions. As an example, an application of the new sensor for 2D pH measurement at a fine-scale in the vivinity of SWI in a freshwater system was demonstrated.

## Results and Discussion

### Design and Ratiometric Imagining of pH

The luminescence CPIPA was selected as the pH-sensitive dye. This indicator dye can absorb green light at 550 nm and emit strong red light with a maximum wavelength of 590 nm. It is insoluble in water, but it is soluble in hydrophobic matrices such as the PVC polymers used in the sensor. Thus, the dye can be easily immobilized, with no apparent leaching in the samples. Intensity-based fluorescence imaging methods have been widely used in previous optical chemical sensing systems^26^,^27^. However, it suffers from various interferences such as non-homogeneity of the light source or sensor layer, background reflection, photobleaching, or leakage of indicator dye for those methods based on one measurement of a single steady fluorescence intensity^7^,^28^. To alleviate these interferences, a well documented ratiometric approach based on two imaging intensities was performed in this system. In contrast to the lifetime-based techniques, this ratiometric referencing technique has been widely used because it is more available andcheaper without sophisticated measuring systems^29^.

**Figure 1:**
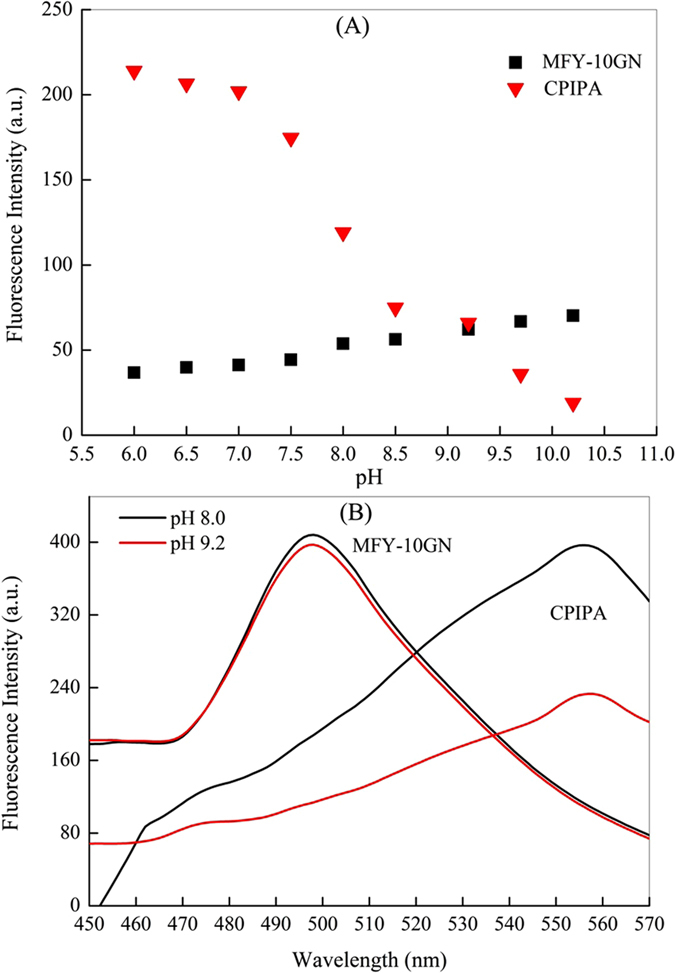
Emission intensities of CPIPA (▾, 550 nm excitation) and those of MFY-10 GN (◾, 389 nm excitation) under different pH (**A**); Excitationspectral data of the indicator dyes CPIPA (λ_excitation-CPIPA_ = 554 nm) and emission spectra of the reference dye MFY-10 GN (λ_emission-MFY-10_ _GN_ = 502 nm) immobilized in the PVC matrix (**B**).

In this study, a ratiometric referencing technique was developed by incorporating pH-sensitive dye CPIPA and another fluorescent dye MFY-10 GN into the sensor films for real-time optical imaging. MFY-10 GN has been demonstrated as an effective pH–insensitive dye owing to its unresponsive character to the pH variation^30^. Upon 550 nm and 389 nm excitation, the dynamic luminescence emission of CPIPA and MFY-10 GN responding to different pH levels are presented in Fig. 1A. The pH indicator CPIPA exhibits an strong pH dependency of its luminescence, while the dye MFY-10 GN showed slightly increase with pH change from 6.0 to 10.2, suggesting its possible use as the internal reference. Moreover, as depicted in Fig. 1B, there was an overlap between the emission spectra of MFY-10 GN and the absorption spectra of the CPIPA, indicating that the two dyes can form a FRET cascade (Förster resonance energy transfer)^31^. The donor MFY-10 GN was excited at 389 nm and transfers its energy to the CPIPA (“the acceptor dye”), emitting subsequent light at 590 nm. Thus, this designed intensity-based referencing type can use ratios of two independent fluorescence signals or images, allowing the simultaneous excitation with a single light source and detection with one photodetector. It should be noted here that, the dye CPIPA exhibited a short Stokes’ shift of 30 nm^32^, which is a challenge for the sensor design due to the interference of the excitation light with the fluorescence emission. MFY-10 GN was an excellent “antenna dye,” which could effectively transfer the emission energy to the absorption indicator (Fig. 1B). The brightness of the emitted luminescence, on one hand, could be dramatically increased by MFY-10 GN; on the other hand, the MFY-10 GN could extend the Stokes’ shift and facilitate the excitation and emission light by light harvesting.

Another key feature of the devised pH sensor film was that the red luminescence was predominantly emitted by the CPIPA, while the blue luminescence was predominantly emitted by MFY-10 GN^30^. This feature enables the sensing of pH using the ratiometrically referenced RGB-imaging method. Fig. S2 depicts the real color pictures derived from the excited sensor films with the apparent color change from red in the acidic region which changed to blue as the pH increased. In the RGB referencing method (see Supplementary information, S1 and Fig. S3), acquired images were split into three independent color channel pictures and analyzed digitally^33^,^34^. Thereafter, the fluorescence intensity ratio (R), defined here as the emission intensity recorded in blue channels (symbolized as the reference) divided by emission intensity recorded in red channels (symbolized as the indicator) was related to pH and was used for calibration. The optodes used in our setup allow imaging of an area of 11.2 × 7.5 cm^2^, equal to a maximum theoretical spatial resolution of approximately 22 × 22 μm.

### Performances of the Sensor for sensing pH

#### Response Time

**Figure 2:**
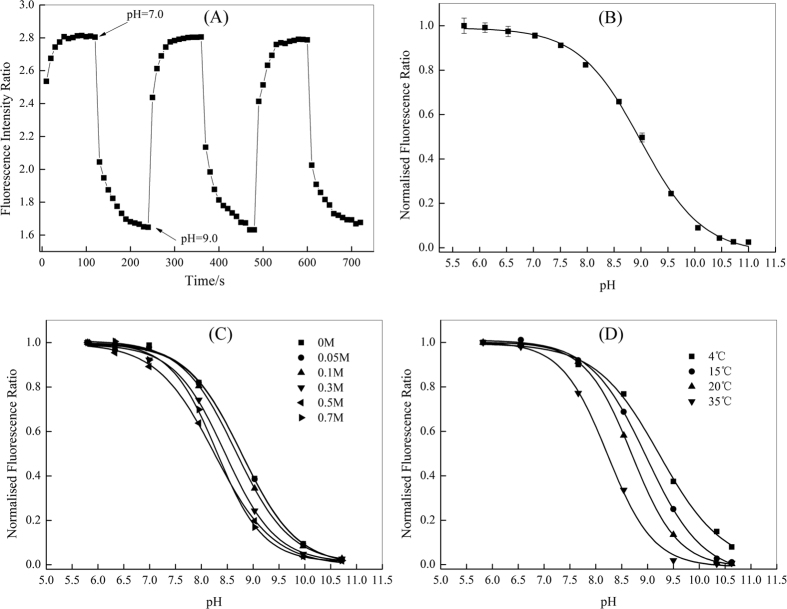
Performances of the sensor for sensing pH. Sensitivity and Response behavior of the sensor when exposed to the pH buffer solution changing from pH 7.0 and pH 9.0 (**A**). The pH response calibration curve based on the RGB read-outreferencing ratiometric method (**B**); error bars represent the standard deviation (SD) of the mean (n = 3). Cross-sensitivity towards varying ionic strength (**C**) and temperature (**D**); the response curves in (**A**,**B**) were obtained at IS = 0.03 M and 25 °C, while all calibration curves presented in (**C**,**D**) were obtained in the laboratory with the same sensing film and with phosphate/NaCl buffer solutions.

One important consideration in the sensor design and characteristics is the response time, with a short response time being desirable due to the rapidly changing pH in some matrixes. The response of the sensor film was evaluated in the pH changing between pH 7.0 and pH 9.0 at a constant room temperature (25 °C) after the buffer equilibration. As shown in Fig. 2A, the response of the sensor is fully reversible and rapid, and requires about 120 s to respond to 90% of the total signals (T_90_) during the transition from pH 7.0 to pH 9.0. Actually, there are about several seconds for the solution equilibrium after the stepwise addition of alkali into the phosphate buffer; thus, the presented sensor has a relatively shorter average response time (T_90_ < 120 s) which is comparable to the values of Zhu *et al*.^5^ and Stahl *et al*.^14^. The response time of the presented sensor is appreciably faster than that of pure CPIPA^32^, which is enough to measure the pH distribution in the sediment or water.

#### Calibration Curve

It is important that the signal ratio is constant over all of the film to get a homogeneous sensor signal^35^. Surface plots of two randomly chosen areas (50 mm × 50 mm) in the intensity image ratio at pH 7.0 and pH 9.0 were used to evaluate the homogeneity of the fluorescence ratio (see Supplementary information, Fig. S4). The calculated ratio signal across the 25 cm^2^ optode area was uniform, with a maximum relative standard deviation (RSD) of ±1.15%. This high uniformity of the sensor clearly demonstrated that the ratiometric referencing technique can largely alleviate concentration-dependent effects within a film such as uneven excitation light and instrumental fluctuation. Therefore, it enables the calibration using an average area approach in contrast to the pixel to pixel calibration.

The sensor’s calibration curve was constructed between normalized fluorescence ratio values (R/R_0_), and pH levels were sigmoidal (S) (Fig. 2B), which has been adopted in other literatures^2^,^30^. It shows that the R values were high for an alkaline environment and decrease when the pH value declines. Ongoing from pH 6.8 to pH 11.5, the signal changes of the normalized R, decreases from 1.0 to 0.0 quantification of pH with a good sensitivity. The *pKa* value of the pH indicator is a crucial parameter for the optical sensor. The apparent *pKa* value (*pKa’*) of the proposed sensor was approximately 9.0, apparently lower than that of immobilized single CPIPA, 10.3^32^. The fitting was highly reliable with a correlation coeffiecient of 0.998. The *pKa’* values of all fluorescent pH indicators is affected by the immobilization matrix and the ionic strength of the medium^2^,^36^. This negative shift may be attributed to the alteration of the acid dissociation caused by the addition of MFY-10GN. Given that the dynamic range of optical pH sensors is usually limited to about *pKa* ± 1.5^36^,^37^, the accurate working range of the proposed sensor extends from pH 7.5 to pH 10.5. The choice of already available pH-sensor for the dynamic range is rather limited. This would closely match the range recorded in previous observations in a variety of lakes or biogeochemical processes^19^,^22^.

#### Cross-sensitivity

The dependency of the calibration curves of the proposed sensor film on ionic strength (IS) at 25 °C is shown in Fig. 2C. The sensor displayed a minimal cross-sensitivity towards IS varying from 0 M to 0.1 M, while the effect at higher IS (>0.3 M) was significant. The alternation of IS from 0 mM to 700 mM was accompanied by the decrease of the *pKa’* values from 9.02 to 8.24. The dependency of the sensor on ionic strength is a well known problem for optical pH sensors when applying them in the shifting IS environment^24^,^38^. However, in the present case, this effect was not critical for pH sensing in the freshwater lakes with IS mostly lowering than 0.1 M.

The temperature cross-sensitivity of the sensor film was investigated from 4 °C to 35 °C (Fig. 2D). There was a special temperature dependency for the sensor, which has been commonly observed in most optical sensors^16^,^39^. This effect can be explained by the reduction of the luminescence quantum yield and the *pKa* values of the indicator caused by the increasing temperature^24^. The cross-sensitivity of the sensor towards temperature can be easily calibrated for the simultaneous monitoring of temperatures.

Dissolved oxygen is an important and ubiquitous fluorescence quencher in the environment^2^. The proposed optical sensor was insensitive to oxygen because the sensor film consisted of a gas-impermeable polymer (PVC), which can prohibit the quenching of the fluorescence emission by the oxygen^11^. Furthermore, it is important to note that the fluorescence intensity of the sensing compound CPIPA in alkaline pH buffers showed no response to the addition of cations such as Na^+^, K^+^ and many polyvalent metal ions^32^,^40^. Accordingly, the proposed pH sensor can be highly selective to pH without interferences from major cations in the natural matrices. Besides, it is appropriate to consider the limitations of this sensor for use in the sediments where Hg (II) is very high (>4.3 ppb), since the Hg (II) dependency of the CPIPA dye is likely to be noticeably observed^40^. However, considering the stronger mineral adsorption and coprecipitation in such alkaline situation, it is expected that the concentration of Hg (II), evidently lower than 0.073ppb (data from our previous investigation for the most freshwater lakes), can hardly affect the sensor response.

### Method Validation and Application

**Figure 3:**
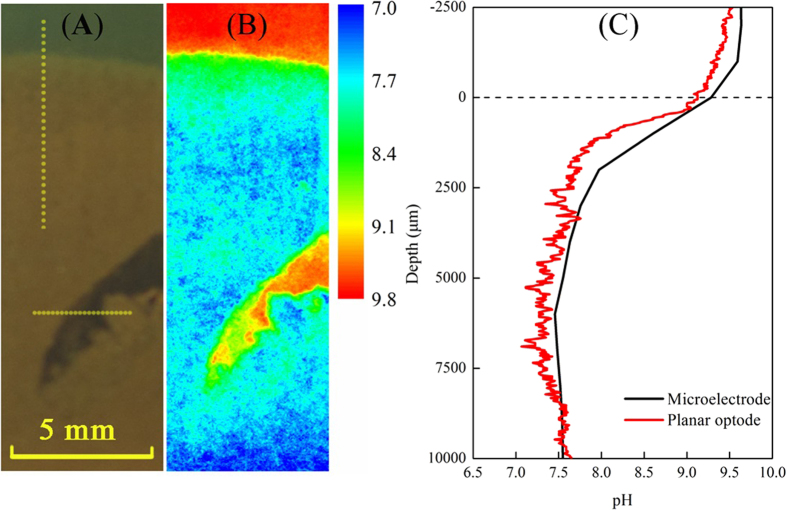
pH distibution pattern across the SWI in the alkaline environment. Side view (**A**) 2D distibution of pH across the SWI in panel (**A**) as elucidated by planar optode (**B**); Comparation of pH profiles measured by the optode planar and miroelectrode. The extracted vertical profile indicated by the yellow dash line acrossing the SWI in panel (**A**), as well as profile (1 mm behind the white line) measured with microelectrode (**C**).

To estimate the method accuracy and precision, the sensor was applied to map pH in the simulated alkaline lacustrine water and sediment by spiking with 1.0 M sodiumm hydroxide solution (see Supplementary information, S2). In Fig. 3, the strong gradient of pH across the SWI was precisely imaged and quantified, revealing that whereas the overlying water pH values were above 9.0, the sediment immediately below was around 7.5 (Fig. 3B). Figure 3C shows the comparison of the data obtained from the proposed sensor and pH microelectrode, and in general very good consistence is observed between two methods, confirming the comparative accuracy and precision of the planar optode measurements. Slight differences between the two profiles methods can be explained by the slight mismatch of their positions in the sediment. Additionally, a time frame series of 2D pH distribution of the artificial burrow acquired by the planar optode was aslo assessed and shown in Fig. S5. It can be concluded that the planar optode allowes the visualization of the rapid pH dynamics of many biogeochemical processes in the alkaline environment.

**Figure 4:**
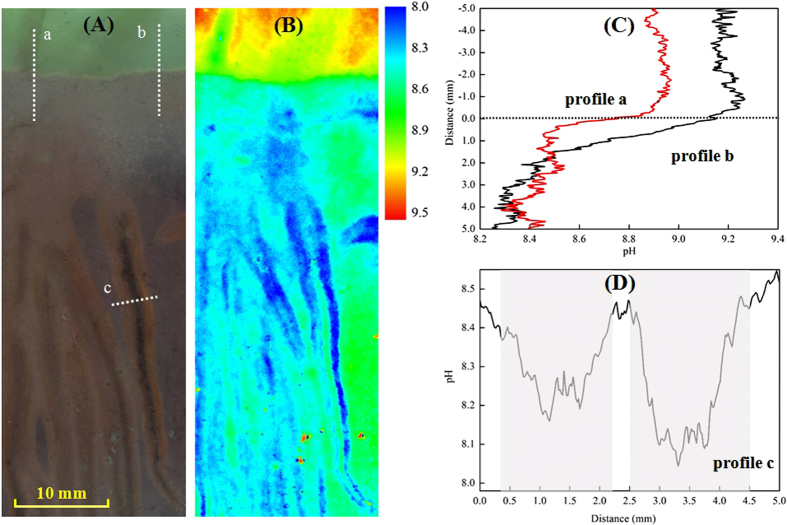
pH distribution patterns in the rhizopshere of *Vallisneria spiralsv*. (**A**) Roots of *Vallisneria spiralsv* visible through the side of the rhizobox. The three white dashed lines (a,b,c) represents the position of the extracted profiles, presented in (**C,D**). (**B**) Corresponsing 2D pH distribution around the roots from*Vallisneria spiralsv* taken after 4 h in the light. The images size is 20 mm × 56 mm. (**C**) Two profiles (a,b) of pH distribution across the sediment and water interface (SWI). The horizontal dashed linerepresents the SWI. (**D**) pH distribution across two single roots from one profile (c), with the rhizosphere zones indicated as shade areas.

Furthermore, the 2D pH distribution in natural sediment-water system associated with *Vallisneria spiralsv*growth was examined. The pH fluorescence image in Fig. 4B shows a virtually heterogeneous distribution with fine structures near the SWI and the rhizosphere of *Vallisneria spiralsv*. High pH (>9.0) in the entire overlying water is observed, which was caused by the photosynthesis^23^. Lower pH values were observed in sediments (<8.6) and especially within the zones around the roots (<8.3). Correspondingly, the profiles of pH variation across the SWI (Fig. 4C) and roots (Fig. 4D) showstrong gradients. Fox example, two 1D pH profiles extracted from the 2D image had a decrease of pH value from 8.9 and 9.2 in the overlying water to around 8.3 at the depth of 4.0 mm below the SWI, respectively (Fig. 4C). One extracted pH profile across two single-root zones showed a W-shape distribution in pH value, reflecting two peak valleys with the corresponding minimums of 8.1 and 8.2 appearing around the rhizosphere (Fig. 4D). The lower pH values in the rhizosphere should be caused by exudation of organc acids such as phenolic and aliphatic compounds^41^. These measurements further verify the feasibility of the presented optical sensor in pH measurement in the natural environments at a high spatial resolution.

### Comparison of Different Planar Optodes Available for Alkaline pH Imaging

Until now, several optodes for the 2D measurement of pH in various matrices have been reported, each has its own advantages and restrictions (listed in Supplementary information, Table S1). Some of these optodes can only partly cover the relevant pH ranges in alkailine^1^,^2^,^14^,^30^ or have an apparent cross-sensitivity towards IS within 0.05 M IS^2^, which are not suitable for application in freshwater sediments. While the proposed sensor enables measurement at pH 7.5 ~ 10.5, a range that is more rarely addressed but is of very importance to applications in those alkaline environmental processes. The optodes based on DHAF or DHFAE in combination with the robust lifetime imaging approaches can provide the increased accuracy with less interferencesfor alkaline pH measurement, but theyarecomparable expensive due to the sophisticated hardware and software^14^,^24^. Compared with the DPPs-based sensors, the presented sensor relies on two fluorescent dyes CPIPA and MFY-10GN, both of which are readily obtained by a simple synthesis procedure of CPIPA or are commercially available at low prices. This sensor retains the advantages of good brightness and excellent photostability. Furthermore, the RGB referencing imaging approach employed in the optode, benefiting from a significantly higher fluorescence brightness, better homogeneity, and shorter response time, can be realized using inexpensive and commercially available digital cameras. All together, the presented sensor combined with the ratiometric imaging approach was more accessible and portable, which can be served well as an inexpensive analytical device.

### Conclusions and Perspectives

The established optical sensor has a relatively wide working range of pH from 7.5 to 10.5. It is tolerant of IS up to 0.1 M with capability of measurement in freshwater sediments. It has a response time of less than 120 s. It is capable of acquiring high spatial resolution of ~22 μm pH images without undue disturbance. Temperature dependence of the sensor on the calibration plots was found and should be taken in consideration. Besides, the presented sensor based on the RGB-color ratiometric imaging method has a significantly higher fluorescence brightness, better homogeneity, faster response time, more portable and inexpensive analytical devices, which is superior to many existing planar optodes. It enables the measurement of fine-scale pH heterogeneity in sedimentsand water relevant to biogenic or abiotic structures such as plant rhizosphere, animal burrow, microbial community and mineral interface. Thereby, application of the proposed pH planar optode may offer a great opportunity for advancing investigations into biogeochemical processes in the aquatic environments.

## Methods

### Materials and Reagents

A sensitive luminophore of the pH sensor presented here is based on a chlorinated Schiff base derivative, chloro phenyl imino propenyl aniline (CPIPA, Supplementary information Fig. S6), which can be synthesized according to Derinkuyu’s method^32^. The coumarin dye MACROLEX^®^ Fluorescent Yellow 10GN (MFY-10GN) was purchased fromBayer MaterialScience. The *p*-chloroaniline and p-Dimethylaminocinnamaldehyde were purchased from *J&K* Chemical, Ltd for the CPIPA synthesis. Polyvinylchloride (PVC), hydrochloric acid, tetrahydrofuran (THF), and dichloromethane (DCM) were of analytical grade, and commercially available from Sinopharm without further purification. Bis-(2-ethylhexyl) phthalate (DOP) was obtained from TCI. Potassium tetrakis-(4-chlorophenyl) borate (PTCPB) was purchased from Sigma Aldrich.

Sodium phosphate, sodium dihydrogen phosphate, sodium hydroxide (Sigma Aldrich) and ultrapure water (18.25 Ω·cm^−1^, Millipore) were used for the preparation of pH buffer solutions. The pH solutions were adjusted to the desired value using MOPS buffers, and the pH of buffer solutions was controlled by a digital pH meter (PHS-3C, INESA Scientific Instrument Company).

### Preparation of the Sensor film

Preparation of the pH sensitive film was modified from previous procedures^30^,^32^. The pH sensing solution was prepared by dissolving 2 mg CPIPA and 3 mg PTCPB in 360 mg of a 1:2 (wt/wt) PVC/DOP mixture. The antenna dye liquid was prepared by dissolving 1 mg MFY-10 GN in 2 mL THF. The indicator and antenna dye liquids were mixed together with vigorous stirring to obtain the “sensing cocktail”. A volume of 200 μL “sensing cocktail” was then uniformly spread onto a 120 μm thick and fully transparent polyethylene terephthalate (PET) film by a self-made knife-coating device. Finally, the film was allowed to evaporate at room temperature for half an hour, and then was protected from light in deionized water prior to use. The thickness of the dried sensor film was about 10 μm. The fluorescent response of this film was stable at least within 10 days (see Supplementary information, Fig. S7).

### Measurement Set-up

Fluorescence excitation and emission spectra as well as response curves of the pH foil were tested on a Shimadzu RF-5301 fluorescence spectrophotometer equipped with a homemade flow-through cell. For all measurements, the temperature was kept constant at 25.0 ± 1.0 °C.

The scheme of the 2D fluorescence imaging system has been widely illustrated elsewhere^1^,^30^. As depicted in Fig. S1, the measurement setup for 2D imaging with a planar optode consists of a sensor layer, containing an analyte-sensitive dye and a reference dye with a thickness of a few micrometers spread on an inert polymeric support. A digital color camera, Canon EOS 600D, equipped with a Sigma 50 mm F2.8 EX DG macro lens, purchased from SIGMA Corporation, Japan, was used for image acquisition and the RGB readout. The camera has an optical resolution of 17.9 million effective pixels (5184 × 3486) and a sensor size of 22.3 × 14.9 mm. Two high-power LED arrays (central wavelength of 385 nm; purchased from SkyBright) were used for excitation of the pH sensitive luminophore. A 450 nm long pass filter OG450 purchased from Schott was used in front of the camera lens to attenuate short-wave light below 450 nm, ensuring that only the emission light of the sensor was recorded.

### Application

Measurements in a natural freshwater sediment and water were conducted to assess the avaibility of the sensor in natural environmental processes. The sensor was deployed in an experimental rhizotron (10 mm × 100 mm × 400 mm) consisted of a transparent acrylic chamber with a front window made of quartz glass. The whole front window (100 mm × 400 mm) was pre-embeded inside a translucentpolycarbonate nuclepore membrane(0.2 μm pore size and 10 μm thickness). Subsequently, the rhizotron were filled with a 15 cm-thickness core sediment and a 30 cm-thickness overlying water collected form Lake Taihu. The selected young *Vallisneria spiral* was then planted in close proximity the polycarbonate nuclepore membrane and cultured under natural light and a temperature of about 25 °C. The rhizotron was kept at an inclination of 30° angle to ensure that roots developed along the front window. After 14 days, the pH sensing film, pre-calibrated with the overlain polycarbonate nuclepore membrane (see Supplementary information, Fig. S3), was gently inserted through the gap between the front window and polycarbonate nuclepore membrane for the imaging application. After 2 hours of equilibration, the camera was fixed onto the front window of the rhizotron and a steady-state pH distribution of the area of interest was measured as mentioned above.

## Additional Information

**How to cite this article**: Han, C. *et al*. High-resolution Imaging of pH in Alkaline Sediments and Water Based on a New Rapid Response Fluorescent Planar Optode. *Sci. Rep*. **6**, 26417; doi: 10.1038/srep26417 (2016).

## Supplementary Material

Supplementary Information
