# Supplementary material for: High-resolution Imaging of pH in Alkaline Sediments and Water Based on a New Rapid Response Fluorescent Planar Optode
Source: Sci Rep. 2016 May 20;6:26417. doi: 10.1038/srep26417 (PMC4873784; doi:10.1038/srep26417)
Supplement: Supplementary Information [file srep26417-s1.pdf]

Supplementary Information

High-resolution Imaging of pH in Alkaline Sediments and Water Based on a New Rapid Response Fluorescent Planar Optode

Chao Han[1], Lei Yao[1], Xu Di[1*], Xianchuan Xie[2], Chaosheng Zhang[3]

[1]State Key Laboratory of Lake Science and Environment, Nanjing Institute of Geography and Limnology, Chinese Academy of Sciences, Nanjing 210008, China.

[2]State Key Laboratory of Pollution Control and Resource Reuse, Center for Hydroscience Research, School of the Environment, Nanjing University, Nanjing 210093, China

[3]GIS Centre, Ryan Institute and School of Geography and Archaeology, National University of Ireland, Galway, Ireland

*Corresponding author: Phone: 86-25-86882209; Fax: 86-25-86882209; E-mail: dxu@niglas.ac.cn.

Contents:

S1: Brief description of ratiometrically referenced RGB-imaging methods for the sensors.

S2: Method Validation.

S3: Calibration Procedure.

Fig.S1: Schematic diagram of the optical imaging system used in this study.

Fig.S2: Real-color picture (RGB image) of the pH sensor array with different pH values.

Fig.S3: Schematic representation of the ratiometrically referenced RGB-imaging

processes.

Fig.S4: 3D distribution of emission fluorensence intensity ratio (R, red/blue) obtained from images reflecting a piece of the sensing film (1×1 cm^2) taken during the sensor calibration at pH 7.0 (top) and pH 9.0 (bottom).

Fig.S5: A time frame series of 2D pH distribution of the artificial burrow acquired by the pH sensor.

Fig.S6: Chemical structure of the indicator dye (CPIPA).

Fig.S7: The fluorescent response of the sensor film after different storage periods.

Table S1. Summary of reported planar optodes available for alkaline pH imaging.

S1: Brief description of ratiometrically referenced RGB-imaging methods for the sensors

The ratiometrically referenced RGB-imaging measurement, which utilizes the intensity ratio between two emission intensities recorded in three independent color channels to enable quantitative imaging of the analyte concentration, can partly overcome some inherent disadvantages of pure fluorescence intensity based imaging. A series of real color RGB images were recorded by the digital camera. The camera parameters were set as follows: 16 bit RAW format, ISO sensitivity 200; aperture 2.8; shutter speed 1/8 s. The acquired images can be opened by Digtial Photo Professional software (DPP, http://www.canon.com.cn/) and then stored with 16 bit TIFF format, afterwards, they were split into the red, green and blue color channels using ImagJ 1.46r software (http://rsb.info.nih.gov/ij/) via Image > Color > Split channels (see **Fig.S4**). Afterwards, the ratiometric images obtained via Process > Image calculator > blue divided by red image (selecting “Create 32 bit float result” in this configuration window). The numerical ratio image values were obtained via Image > Transform >

Image to results, and further fitted with the previously obtained calibration curve.

S2. Method Validation

A laboratory experiment was conducted to test the performance of the sensor for pH imaging of the sediment. The sediments and bottom water were collected from Lake Taihu. The fresh sediments were sieved (1 mm mesh) and mixed to obtain the homogenized subsamples, and then were transferred into a rectangular microcosm tank and incubated in the dark at room temperature for two weeks. A second box (200×100×100 mm), with open top and bottom, was prepared with a quartz window. Before the second box was inserted into the culture sediment, a calibrated sensor film was stuck to the inside of the quartz window of the box without the presence of bubbles. A well-established pH microelectrode having a tip diameter of 30-50 μm and response time of 2 seconds was used to measure pH profiles next to the planar optode for comparison. The microelectrode were moved and positioned with an accuracy of ~1 mm driven by a Tesa Hite 300 micromanipulator.

S3. Calibration Procedure

The sensor calibration was accomplished using a 40 × 40 mm sensor film mounted into a black PMMA aquarium (100 mm×100 mm×50 mm) with a removable front window (made of quartz glass, 100mm×100 mm). Prior to the calibration procedure, a duplicate of the used pH sensor film was attached tightly to the inside of the window, taking care to exclude air bubbles. The film was immobilized with small pieces of waterproof tape. The calibration aquarium was then filled alternately with different standard buffers ranging from pH 5.6 to pH 11.0, and the respective fluorescence images (RAW format) of the planar optode were acquired with the optical set-up in

Fig. S1. The camera was positioned perpendicularly to the acrylic plane, and the LEDs were orientated at a 30° angle relative to the box. It should be noted that the whole imaging process was performed in the dark to avoid any external light interference. Calibration of the planar optode was done in water before and after the experiments, and no change in the sensor response was observed.

The normalized pH calibration ranging from 6.5 to 10.5 can be fitted by the Boltzmann function with four parameters (Eq.1): (Schroder et al., 2005; Rudolph et al., 2013)

$$\frac{R}{R_0} = \frac{m1 - m2}{1 + \exp\left(\frac{pH - pKa'}{p}\right)} + m2 \qquad (1)$$

where R and R_0 are calculated ratios at varying pH values and at the lowest pH value used during the calibration (pH 6.5), respectively; *m1, m2, pKa', * and *P* are the numerical coefficients describing the initial value (*m1*), the final value (*m2*), the point of inflection (*pKa'*), and the width (*P*) of the S curve.

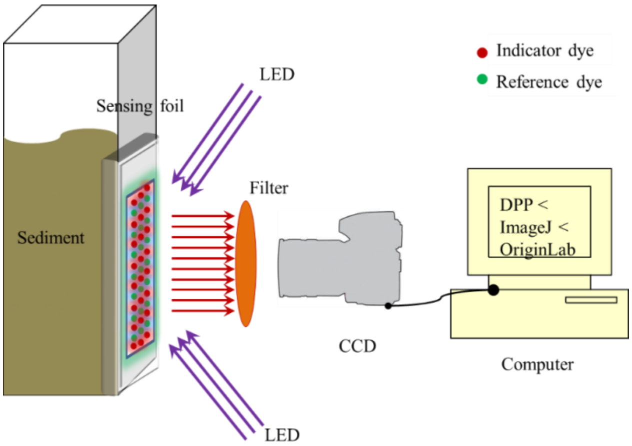

Fig.S1 Schematic diagram of the optical imaging system used in this study.

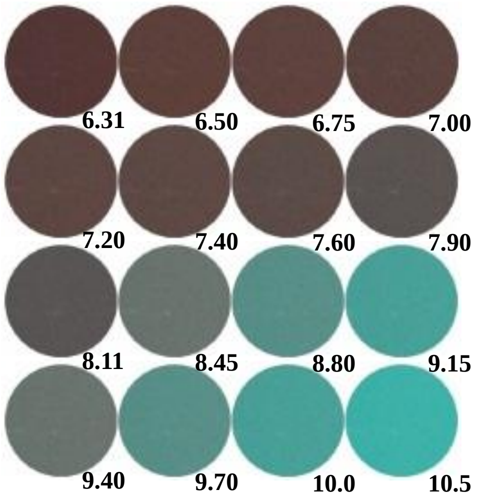

Fig.S2 Real-color picture (RGB image) of the pH sensor array with different pH values.

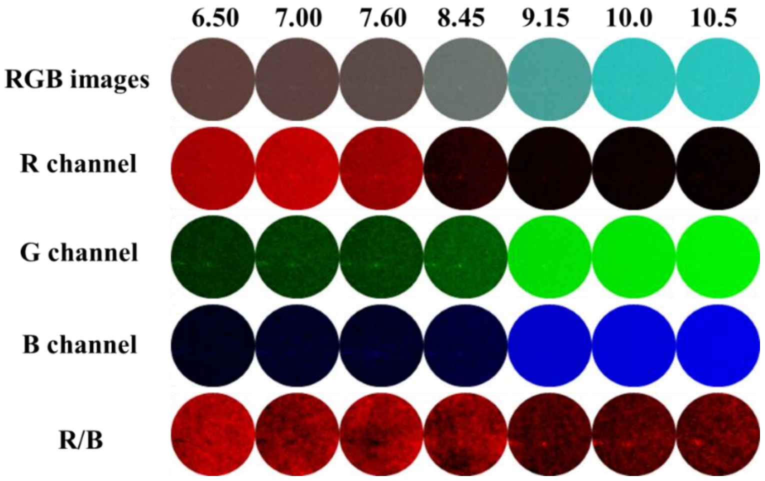

Fig.S3 Schematic representation of the ratiometrically referenced RGB-imaging processes.

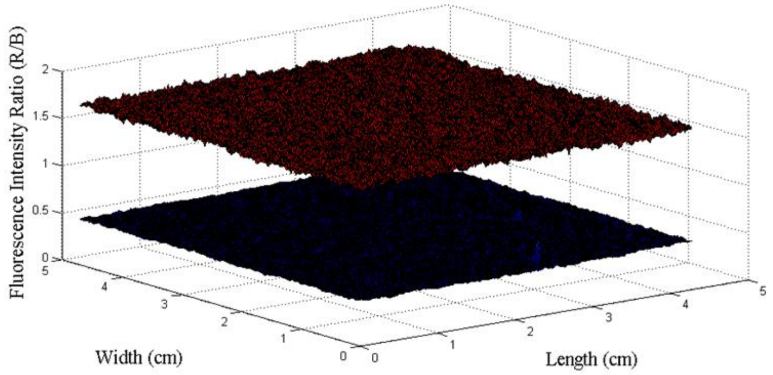

Fig. S4 3D distribution of emission fluorensence intensity ratio (R, red/blue) obtained from images reflecting a piece of the sensing film (1×1 cm^2) taken during the sensor calibration at pH 7.0 (top) and pH 9.0 (bottom).

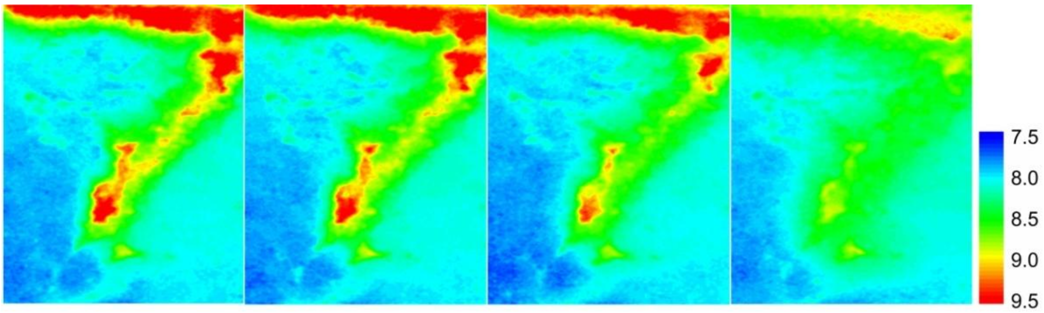

Fig.S5 A time frame series of 2D pH distribution of the artificial burrow acquired by the pH sensor. Each image was acquired every 5 min in the darkness. An artificial burrow was constructed by a syringe needle. Four maps monitoring pH dynamics of the burrow were taken at 5 min intervals after the addition of 1.0 M sodiumm hydroxide solution into the vicinity of the artificial burrow with the syringe, showing the temporal pH dynamics. As seen, the boundary between water and sediment zones as well as the burrow structure in the microcosm were clearly visible. The average pH values around the small burrow structure decreased from around 9.5 to below 8.5 during the imaging series.

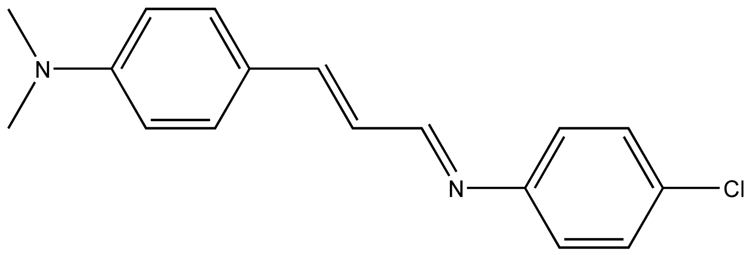

Fig.S6 Chemical structure of the indicator dye (CPIPA)

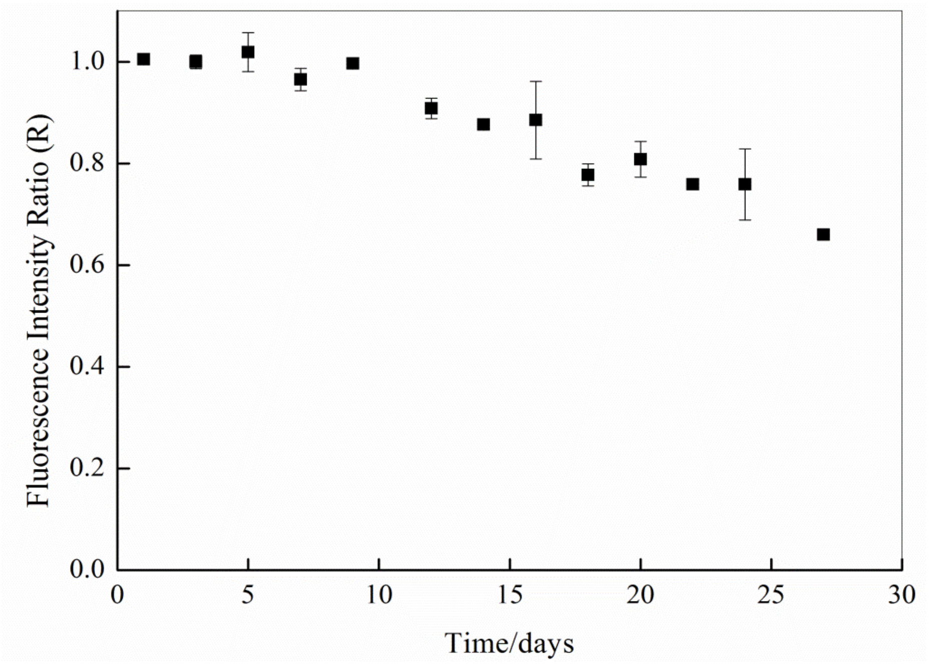

Fig.S7 The fluorescent response of the sensor film after different storage periods. All measurements were taking with a phosphate buffer solution adjusted to pH 9.5 (IS: 0.03 mM, T: 25 ℃) . The optical setup was the same as used throughout the work. Error bars represent the standard deviation (SD) of the mean (n=3).

Table S1. Summary of reported planar optodes available for alkaline pH imaging.

Indicator	Working Range	Response Time (s)	*pKa*	Imaging Approach	Comments	Ref
lipophilic HPTS	6~9	60 s	7.47	Intensity-based RGB referencing method	The lipophilic indicators (HPTS$(OA)_3$) and MFY-10GN dyes were immobilized into polyurethane hydrogel.Excellent photostability and brightness; simple and inexpensive; appropriate for a wide range of environmental samples.	30
hydrophilic HPTS	5.5~8.6	<120 s	7.06	Intensity-based fluorescence emission ratiometric	Amino-modified HTPS dye was covalently conjugated into poly-N-isopropylacrylamide polymer chain and bound on the surface of PVA membrane. Tedious and time-consuming; highly sensitivity towards IS within 0.05 M.	2
HPTS	5.4~7.4	< 5 s	6.4	Intensity-based pixel ratio	HPTS dye physically adsorbed onto a cellulose acetate. Relatively soft and readily damaged in common applications.	12
lipophilic carboxyfluorescein derivatives(DHFAE[a],DHFA[b])	7.3~9.3 (DHFAE) 7.2~9.2 (DHFA)	< 230 s	~8.3	Time domain dual-lifetime referencing (t-DLR)	Immobilized the fluorescence DHFAE/DHFA and the phosphorescent ruthenium compounds into the hydrogel matrix. Better accuracy and signal stability; low cross-sensitivity towards IS; requirements for expensive imaging devices.	14,24
DPPs[c]	5.0~12.0	< 2 s	8.0~11.0	Intensity-based RGB referencing method	A wider operation pH range can be tuned by appropriate selection of synthesized DPPs dyes and polymer hydrogels matrices. Relatively time-consuming synthesis and tedious purifying with modest yields (13-22%).	25

CPIPA	7.52~10.52	< 90 s	9.02	Intensity-based RGB referencing method	Synthesized by a single, simple reaction step without purifying with nearly 100% yields. Excellent brightness and photostability; inexpensive and more accessible imaging scheme.	This work

[a]2´,7´-dihexyl-5(6)-Noctadecyl-carboxamidofluorescein ethyl ester (DHFAE), [b]2´,7´-dihexyl-5(6)-N-octadecyl-carboxamidofluorescein (DHFA), [c]1,4-diketopyrrolo-[3,4-c]pyrrole derivatives (DPPs).
